# An efficient genome-wide association test for multivariate phenotypes based on the Fisher combination function

**DOI:** 10.1186/s12859-015-0868-6

**Published:** 2016-01-05

**Authors:** James J. Yang, Jia Li, L. Keoki Williams, Anne Buu

**Affiliations:** School of Nursing, University of Michigan, Ann Arbor, Michigan USA; Department of Public Health Sciences, Henry Ford Health System, Detroit, Michigan USA; Center for Health Policy and Health Services Research, Henry Ford Health System, Detroit, Michigan USA; Department of Systems, Populations and Leadership, University of Michigan, Ann Arbor, Michigan USA

**Keywords:** Genome-wide association study, Fisher combination function, Multivariate permutation, Principal component analysis

## Abstract

**Background:**

In genome-wide association studies (GWAS) for complex diseases, the association between a SNP and each phenotype is usually weak. Combining multiple related phenotypic traits can increase the power of gene search and thus is a practically important area that requires methodology work. This study provides a comprehensive review of existing methods for conducting GWAS on complex diseases with multiple phenotypes including the multivariate analysis of variance (MANOVA), the principal component analysis (PCA), the generalizing estimating equations (GEE), the trait-based association test involving the extended Simes procedure (TATES), and the classical Fisher combination test. We propose a new method that relaxes the unrealistic independence assumption of the classical Fisher combination test and is computationally efficient. To demonstrate applications of the proposed method, we also present the results of statistical analysis on the Study of Addiction: Genetics and Environment (SAGE) data.

**Results:**

Our simulation study shows that the proposed method has higher power than existing methods while controlling for the type I error rate. The GEE and the classical Fisher combination test, on the other hand, do not control the type I error rate and thus are not recommended. In general, the power of the competing methods decreases as the correlation between phenotypes increases. All the methods tend to have lower power when the multivariate phenotypes come from long tailed distributions. The real data analysis also demonstrates that the proposed method allows us to compare the marginal results with the multivariate results and specify which SNPs are specific to a particular phenotype or contribute to the common construct.

**Conclusions:**

The proposed method outperforms existing methods in most settings and also has great applications in GWAS on complex diseases with multiple phenotypes such as the substance abuse disorders.

## Background

In the past decade, genome-wide association studies (GWAS) have produced rich single-nucleotide polymorphism (SNP) data available to researchers. Among them, the large scale studies including the HapMap project [1] and the 1000 Genomes project [2] have provided publicly accessible databases of reference ancestral populations for imputation and quality control purposes. The idea of GWAS is to conduct fast SNP-based association tests to scan the whole genome using case-control samples. Yet, many complex diseases such as mental health disorders may have multiple phenotypic traits with continuous outcomes [3]. This pleiotropy in complex traits [4] provides several potential advantages to the direct modeling of pleiotropic associations. First, a model search for loci that are simultaneously associated with multiple phenotypes would likely have higher power than a model search that only considers each phenotype individually. Second, more exact modeling may yield more accurate prediction of either or both phenotypes. Third, pleiotropic genes may tend to have a more central role in the relevant functional pathways.

Existing statistical methods for complex diseases with multivariate phenotypes can be categorized into three types of approaches. The first approach is to conduct a GWAS for each marginal phenotype and then aggregate the results. The major issue with this approach is that it does not make use of the correlation structure among phenotypes. The second approach is to summarize multiple phenotypic traits into a composite score and then conduct a GWAS on the score. This approach, however, may have difficulty in identifying proper summary scores. The third approach involves multiple phenotypic traits simultaneously. Thus, it may gain power as well as avoid the issue of multiple testing. However, it is based on stronger assumptions that may not be satisfied in some practical settings.

In this study, we provide a comprehensive review of existing statistical methods for conducting GWAS on complex diseases with multiple phenotypic traits. We also propose a new statistical method based on the Fisher combination function. The performance of competing methods is evaluated by a simulation study. In order to demonstrate applications of the proposed method, we conduct statistical analysis on the database of the Study of Addiction: Genetics and Environment (SAGE).

## Methods

Let *X*_*i*_(=0,1,2) be the number of reference alleles corresponding to a candidate SNP and ***Y***_*i*_=(*Y*_*i*1_,…,*Y*_*im*_)^′^ be the measures of multiple phenotypes for the individual *i*. In this study, we conduct a comprehensive review of existing statistical methods that can be used to test the association between *X*_*i*_ and ***Y***_*i*_.

### Existing methods

#### Multivariate analysis of variance (MANOVA)

When the phenotype is univariate, we can use the one-way analysis of variance (ANOVA) with three levels of the genotype for GWAS. When we have correlated multivariate phenotypic traits, the natural extension of the one-way ANOVA is the one-way multivariate analysis of variance (MANOVA) [5]. Similar to ANOVA, MANOVA tests the equality of mean phenotypic vectors by comparing the within genotypes and between genotypes variance-covariance matrices. The strength of MANOVA is that the multivariate normal distribution provides many good statistical properties for testing and estimation [6]. However, in practice, multivariate phenotype data are very unlikely to meet the multivariate normal assumption. Furthermore, MANOVA is most powerful when the phenotypes are negatively correlated and yet this situation is also unlikely in practice, especially when the number of phenotypes is larger than 2. With respect to its relevant applications, this method has been used in GWAS on dose-response [7] and facial morphology [8].

#### Principal component analysis (PCA)

The principal component analysis (PCA) [9, 10] is another classical statistical method for multivariate analysis. The primary objective of PCA is to find a small set of linear combinations of the original variables (i.e. principal components) that account for the most variability in the original variables. Thus, it can be employed to reduce the dimension of multivariate phenotypes. The PCA has been used in gene-based studies to increase the power of statistical testing [11, 12]. Furthermore, He et al. [13] has used PCA to combine four highly correlated obesity phenotypes for a whole genome linkage scan. When the phenotypes are highly correlated, the first principal component (corresponding to the largest eigenvalue) contains most information about the phenotype data. Thus, testing the association between a SNP and the first principal component is a commonly adopted approach to effectively change the multivariate setting associated with multiple phenotypes in GWAS to the univariate setting (e.g. Zhang et al. [14] and Karasik et al. [15]). In this study, we investigate the statistical properties of this approach.

#### Generalized estimating equations (GEE)

The method of generalized estimating equations (GEE) [16] was developed for analyzing correlated multivariate outcomes primarily from longitudinal studies. It can be applied to test the association between a candidate SNP and multivariate phenotypes. The GEE only requires specification of the link function and the working correlation matrix. The former depends on the measurement scale of the outcomes (e.g. the identify link for continuous outcomes). The latter assumes the correlation structure among multivariate outcomes. The estimation of GEE is usually robust against this assumption. GEE was widely used in GWAS. For example, GEE was proposed as one of the multivariate approaches in Solovieff et al. [4]. For another example, Liu et al. [17] proposed to use GEE for bivariate association analyses for the mixture of continuous and binary traits. However, to the best of our knowledge, none of the existing studies have conducted simulations to investigate whether GEE can control the type I error rate when multivariate traits are involved in GWAS. To fill in this knowledge gap, we conduct a simulation study to examine the statistical properties of this approach. In this study, we only consider the identity link because we are mainly interested in continuous phenotypic traits. We also assume the working correlation matrix to be compound symmetry because it only requires us to estimate one additional parameter.

#### Trait-based association test involving the extended Simes procedure

Recently, van der Sluis et al. [18] developed a trait-based association test involving the extended Simes procedure (TATES). The TATES calculates a global *p*-value based on individual *p*-values of association tests for marginal phenotypes. Specifically, for *m*-variate phenotypic traits, one can conduct *m* tests of the association between a candidate SNP and each marginal phenotypic trait and derive *mp*-values: *p*_1_,…*p*_*m*_. Let *p*_(1)_,…*p*_(*m*)_ be the ordered *p*-values from the smallest to the largest. The Simes multiple procedure declares significance between a SNP and multivariate phenotypic traits at the *α* level if any of the *p*-values satisfy *p*_(*j*)_<*jα*/*m* [19]. Hence, the global *p*-value based on the Simes procedure is *p*_traits_= min{*mp*_(*j*)_/*j,j*=1,…,*m*}. The TATES improves this procedure by replacing *m* and *j* with the effective number of independent traits, *m*_*e*_ and *j*_*e*_, which are estimated from the eigenvalues of the correlation matrix [20, 21]. Since *m*_*e*_≤*m*, this new adjusted global *p*-value, defined as *p*_traits_= min{*m*_*e*_*p*_(*j*)_/*j*_*e*_,*j*=1,…,*m*} is smaller than the Simes global *p*-value. Therefore, the TATES is more powerful than the Simes. A simulation study also showed that the TATES is more powerful than MANOVA. In this study, we conduct a comprehensive simulation study to compare this method with not only the classical methods reviewed above but also the proposed methods.

### Proposed methods

#### The methods based on the Fisher combination function

Combining independent tests of significance to form a join statistic has been used as an alternative approach to tackling complex multivariate location problems [22]. This approach is quite popular in practice because it is much easier to develop a univariate association test statistic than a multivariate association test statistic. Birnbaum [23] discussed various combination functions among which the Fisher combination function has been proven to be asymptotically Bahadur optimal [24, 25]. Thus, we focus on the Fisher combination function in this study.

#### Fisher combination test with the independence assumption

Based on the Fisher combination test [22], to test the association between a SNP and multivariate phenotypic traits, we only need to test the association between the SNP and each marginal phenotypic trait individually. Thus, for *m*-multivariate phenotypes, we have *m* marginal *p*-values: *p*_1_,…,*p*_*m*_. The Fisher combination statistic is defined as

(1) $$ T = \sum_{j=1}^{m} -2\log(p_{j}).   $$

*T* is used to infer the association between the SNP and multivariate phenotypic traits. When the marginal *p*-values are independent, the statistic *T* follows a chi-squared distribution with 2*m* degrees of freedom so the *p*-value of *T* can be obtained straightforwardly. In reality, however, the phenotypic traits are always correlated so the chi-squared distribution with 2*m* degrees of freedom tends to underestimate the variance of the *T* statistic. The resulting chi-squared test is, therefore, too liberal.

#### The permutation method

Because of the negative consequence of the independence assumption, it is desirable to conduct the Fisher combination test without the assumption. Ideally, we could calculate the exact *p*-value of the *T* statistic in Eq. (1) using the permutation method, which does not require the unrealistic assumption and also controls for the type I error [26]. Yet, the permutation method is a very time-consuming procedure, particularly in a genome-wide context. Thus, an improvement in computational efficiency is warranted.

#### The proposed efficient method

For correlated phenotypic traits, *T* is the sum of dependent chi-squared statistics. Brown [27] and Yang [28] have shown that, under the null hypothesis of no association between a SNP and multivariate phenotypic traits, the distribution of *T* statistic follows a scale chi-squared distribution $(\gamma {\chi _{v}^{2}})$, or equivalently, a gamma distribution with the shape parameter *v*/2 and the scale parameter 2*γ*. Therefore, to calculate the global *p*-value of *T* statistic, we only need to estimate the parameters *v* and *γ*. Suppose that the mean of *T* is *μ* and the variance of *T* is *σ*^2^. Using the first and second moments of *T*, the values of *v* and *γ* can be calculated as *v*=2*μ*^2^/*σ*^2^ and *γ*=*σ*^2^/(2*μ*). The following are technical details of the derivation of the mean and variance of *T* statistic when the marginal *p*-values are based on two-sided tests (see Brown [27] and Yang [28] for the case of one-sided marginal tests):

Without loss of generality, we assume that the association test statistic for the *j*th phenotypic trait is *z*_*j*_ where *j*=1,…,*m*. The corresponding two-sided *p*-value is defined as *p*_*j*_=2*Φ*(−|*z*_*j*_|), where *Φ* is the standard Gaussian distribution function. Under the null hypothesis of no association between a SNP and multivariate phenotypic traits, the distribution of *T* statistic is approximated by a Gaussian distribution with the mean of *T* as

$$ \mu = E[T] = 2m $$

and the variance of *T* as

$$\begin{array}{@{}rcl@{}} \sigma^{2} &=& \text{Var}[T] \\ & = &\text{Var} \left\{\sum_{j=1}^{m} -2\log(p_{j})\right\}\\ & = & \sum_{j=1}^{m} \text{Var}\{-2\log(p_{j})\} \! +\! \sum_{j \ne k} \text{cov}\{-2\log(p_{j}), \! -2\log(p_{k}\!)\}\\ & = & 4m + \sum_{j \ne k} \text{cov}\{-2\log(p_{j}), -2\log(p_{k})\}. \end{array} $$

Therefore, in order to calculate the variance of *T*, we need to calculate the covariance for each pair (*j,k*) which can be expressed as

$${\fontsize{8.5pt}{9.3pt}\selectfont{ \begin{aligned} {}\lefteqn{\text{cov}\{-2\log(p_{j}), -2\log(p_{k})\}}\\ {}&=& E\{[-2\log(p_{j})] [-2\log(p_{k})]\} - E\{-2\log(p_{j})\}E\{-2\log(p_{k})\}\\ {}&=& 4\int_{-\infty}^{\infty}\int_{-\infty}^{\infty}\log\{2\Phi(-|z_{j}|)\} \log\{2\Phi(-|z_{k}|)\}dF(z_{j},z_{k}) - 4, \end{aligned}}} $$

where *F* is the standard bivariate Gaussian distribution. Let

$$\delta_{jk}=\text{cov}\{-2\log(p_{j}), -2\log(p_{k})\}. $$

**Fig. 1:**
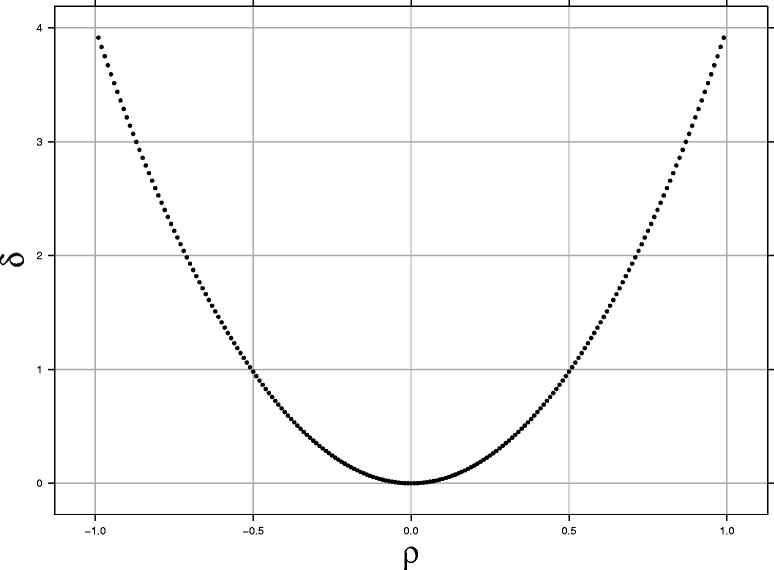
The relationship between the covariance *δ* (*y*-axis) and the correlation *ρ* (*x*-axis)

Thus, *δ*_*jk*_ is a function of the correlation between *z*_*j*_ and *z*_*k*_: *ρ*_*j,k*_. We explore the relationship between *δ*_*jk*_ and *ρ*_*j,k*_ by calculating *δ*_*jk*_ numerically for the values of *ρ*_*j,k*_ from -0.99 to 0.99 with the step of 0.01. The results are shown in Fig. 1. Since the curve of Fig. 1 is a convex curve symmetric about the *y*-axis, we can approximate the relationship between *δ*_*jk*_ and *ρ*_*j,k*_ using a tenth-order polynomial:

(2) $$ \delta_{jk} \doteq c_{1}\rho_{j,k}^{2}+c_{2}\rho_{j,k}^{4}+c_{3}\rho_{j,k}^{6} +c_{4}\rho_{j,k}^{8}+c_{5}\rho_{j,k}^{10}.   $$

Using the adapt function in the R package fCopulae [29], we obtained the following estimates: *c*_1_=3.9081,*c*_2_=0.0313,*c*_3_=0.1022,*c*_4_=−0.1378 and *c*_5_=0.0941; the maximum residual was less than 0.0001.

To estimate *δ*_*jk*_ in Eq. (2) accurately, we have taken two steps to remove potential biases. First, since the sample correlation $\hat {\rho }_{\text {\textit {j,k}}}$ is not an unbiased estimator of *ρ*_*j,k*_ [30], we estimate *ρ*_*j,k*_ by the bias-corrected sample correlation $\hat {r}_{\text {\textit {j,k}}}$:

(3) $$ \hat{r}_{j,k} = \hat{\rho}_{j,k}\left(1+ \frac{1-\hat{\rho}_{j,k}^{2}}{2(n-3)} \right),   $$

where *n* is the samples size used to calculate $\hat {\rho }_{\text {\textit {j,k}}}$. Now, let’s define the right hand side of Eq. (2) as

$$f(r) = c_{1} r^{2} + c_{2}r^{4} + c_{3} r^{6} + c_{4}r^{8}+c_{5} r^{10}. $$

The estimate $f(\hat {r}_{\text {\textit {j,k}}})$ is still a biased estimator of *δ*_*jk*_. Thus, we propose a second step to remove the bias. Using the Taylor series expansion, we can estimate the bias as

$$\frac{c_{1}}{n}\left(1-\hat{r}_{j,k}^{2} \right)^{2}. $$

Therefore, the proposed unbiased estimator of *δ*_*jk*_ is

(4) $$ f(\hat{r}_{j,k}) - \frac{c_{1}}{n}\left(1-\hat{r}_{j,k}^{2} \right)^{2}.   $$

Hence, based on Eqs. (3) and (4), the variance of *T* can be estimated as

$$\sigma^{2} = \text{Var}(T) \doteq 4m + \sum_{j \ne k} \left(f(\hat{r}_{j,k}) - \frac{c_{1}}{n}\left(1-\hat{r}_{j,k}^{2} \right)^{2} \right). $$

Given the proposed estimators of *μ* and *σ*^2^, the global *p*-value of *T* statistic can be computed efficiently using the gamma distribution function as follows:

$$\text{the global } p\text{-value} = 1- \Gamma\left(\mu^{2}/\sigma^{2}, \sigma^{2}/\mu\right), $$

where *Γ*(*v*/2,2*γ*) is the gamma distribution function with the shape parameter *v*/2 and the scale parameter 2*γ*.

In this study, we compare two alternative methods to calculate $\hat {\rho }_{\text {\textit {j,k}}}$: the Pearson sample correlation coefficient and the rank correlation coefficient of Kendall’s *τ*. Kendall and Gibbons [31] have shown the relation between *ρ* and *τ* as

$$\rho= \sin\left(\frac{\pi\tau}{2}\right). $$

Thus, we can use Kendall’s *τ* to derive $\hat {\rho }_{\text {\textit {j,k}}}$ as $\sin \left (\frac {\pi \hat {\tau }_{\text {\textit {j,k}}}}{2}\right)$. In the simulation study, we evaluate the robustness of Kendall’s *τ* in comparison to Pearson’s sample correlation coefficient.

## Results

### Simulation study

We conducted a simulation study to evaluate the performance of the four methods reviewed (MANOVA, PCA, GEE, and TATES) as well as the methods based on the Fisher combination function. In the simulation, we adopted four different approaches to calculating the *p*-value of the Fisher combination test:

1. FC-$\chi _{2m}^{2}$: the chi-squared distribution with 2*m* degrees of freedom under the independence assumption.
2. FC-Permutation: the permutation method based on 1000 permutes.
3. FC-Pearson: the proposed method with the correlation $\hat {\rho }_{\text {\textit {j,k}}}$ being estimated by the Pearson’s sample correlation coefficient.
4. FC-Kendall: the proposed method with $\hat {\rho }_{\text {\textit {j,k}}}$ being estimated by the Kendall’s *τ*.

#### Simulation configurations

For each subject, the relationship between the SNP, *x*, and the multivariate phenotypes, ***Y***, was defined as

$${\boldsymbol{Y}} = x{\mathbf{\beta}} + {\mathbf{\epsilon}}, $$

where **β** is the effect of the SNP on the phenotypes and **ε** is the error term. In this simulation study, we simulated *x* based on a minor allele frequency of 0.25. We evaluated the performance of competing methods under three different settings of effect sizes: **Null hypothesis (no effects)β**=(0,0,0,0,0)^′^. **Moderate equal effect sizesβ**=(0.3,0.3,0.3,0.3,0.3)^′^. **Varied effect sizesβ**=(0.1,0.2,0.3,0.4,0.5)^′^.

For the error term, we considered two cases. In the first case, the error term was simulated from a multivariate normal distribution with the mean **0** and the variance-covariance ***Σ***, which is a compound symmetry matrix with the value of 1 on the diagonal and the value of *ϱ*=0,0.25,0.5, or 0.75 on the off-diagonal. In the second case, the error term was simulated from a mixture of two multivariate normal distributions: 90 % from the same multivariate normal distribution in the first case and 10 % from the multivariate normal distribution with the mean **0** and the variance-covariance matrix 5***Σ***. The purpose of the second case was to simulate long tailed distributions of phenotypic traits which are common in real data. We generated simulated data of 100 subjects under each configuration. In addition, each configuration was repeated 10,000 times. The nominal type I error rate was set at 0.05 and the power was calculated as the proportion of *p*-values less than 0.05.

#### Simulation results

Table 1 presents the simulation results when the multivariate phenotypes come from a multivariate normal distribution with the value of the correlation *ϱ* varied from 0 to 0.75. The numbers in each cell are the mean (standard deviation) of the indicator variable for *p*-value <0.05 among the 10,000 replications. The top panel corresponds to the case of **β**=(0,0,0,0,0)^′^ (i.e. when the null hypothesis is true) and thus can be used to evaluate if each of the competing methods was able to control the type I error. The results indicate that GEE and the Fisher combination test with $\chi _{2m}^{2}$ did not control the type I error rate when *ϱ*>0 while all the other methods did quite well under all values of *ϱ*. We, thus, did not find it meaningful to further compare these two methods with the other methods in terms of the statistical power.

**Table 1:** Simulation results when the multivariate phenotypes come from a multivariate normal distribution

*ϱ*	MANOVA	PCA	GEE	TATES	FC-$\chi _{2m}^{2}$	FC-Permutation	FC-Pearson	FC-Kendall
	**β**=(0,0,0,0,0)^′^							
0	0.0477	0.0514	0.0109	0.0487	0.0468	0.0455	0.0455	0.0451
	(0.0021)	(0.0022)	(0.0010)	(0.0022)	(0.0021)	(0.0021)	(0.0021)	(0.0021)
0.25	0.0477	0.0499	0.0763	0.0498	0.0631	0.0488	0.0482	0.0477
	(0.0021)	(0.0022)	(0.0027)	(0.0022)	(0.0024)	(0.0022)	(0.0021)	(0.0021)
0.5	0.0477	0.0496	0.1518	0.0506	0.0942	0.0473	0.0482	0.0484
	(0.0021)	(0.0022)	(0.0036)	(0.0022)	(0.0029)	(0.0021)	(0.0021)	(0.0021)
0.75	0.0477	0.0496	0.2202	0.0494	0.1263	0.0467	0.0489	0.0485
	(0.0021)	(0.0022)	(0.0041)	(0.0022)	(0.0033)	(0.0021)	(0.0022)	(0.0021)
	**β**=(0.3,0.3,0.3,0.3,0.3)^′^							
0	0.7595	0.5679	0.9333	0.7359	0.9067	0.9058	0.9047	0.9040
	(0.0043)	(0.0050)	(0.0025)	(0.0044)	(0.0029)	(0.0029)	(0.0029)	(0.0029)
0.25	0.4086	0.7075	0.8570	0.6406	0.8076	0.7748	0.7749	0.7749
	(0.0049)	(0.0045)	(0.0035)	(0.0048)	(0.0039)	(0.0042)	(0.0042)	(0.0042)
0.5	0.2655	0.5295	0.8113	0.5668	0.7411	0.6314	0.6420	0.6421
	(0.0044)	(0.0050)	(0.0039)	(0.0050)	(0.0044)	(0.0048)	(0.0048)	(0.0048)
0.75	0.2011	0.4144	0.7827	0.4949	0.6927	0.5169	0.5272	0.5278
	(0.0040)	(0.0049)	(0.0041)	(0.0050)	(0.0046)	(0.0050)	(0.0050)	(0.0050)
	**β**=(0.1,0.2,0.3,0.4,0.5)^′^							
0	0.8550	0.6646	0.9272	0.8731	0.9457	0.9454	0.9448	0.9445
	(0.0035)	(0.0047)	(0.0026)	(0.0033)	(0.0023)	(0.0023)	(0.0023)	(0.0023)
0.25	0.6334	0.7243	0.8500	0.8237	0.8864	0.8604	0.8631	0.8621
	(0.0048)	(0.0045)	(0.0036)	(0.0038)	(0.0032)	(0.0035)	(0.0034)	(0.0034)
0.5	0.6203	0.5437	0.8043	0.7758	0.8283	0.7252	0.7334	0.7333
	(0.0049)	(0.0050)	(0.0040)	(0.0042)	(0.0038)	(0.0045)	(0.0044)	(0.0044)
0.75	0.8177	0.4227	0.7756	0.7512	0.7721	0.5821	0.5942	0.5941
	(0.0039)	(0.0049)	(0.0042)	(0.0043)	(0.0042)	(0.0049)	(0.0049)	(0.0049)

The three different effect sizes are: no effect **β**=(0,0,0,0,0)^′^; moderate effects **β**=(0.3,0.3,0.3,0.3,0.3)^′^; and varied effects **β**=(0.1,0.2,0.3,0.4,0.5)^′^. The correlation between genes is *ϱ* ranging from 0 to 0.75. The competing methods are MANOVA (Multivariate analysis of variance), PCA (Principal component analysis), GEE (Generalized estimating equations), TATES (Trait-based association test involving the extended Simes procedure), FC-$\chi _{2m}^{2}$ (the chi-squared distribution with 2*m* degrees of freedom under the independence assumption), FC-Permutation (the permutation method based on 1,000 permutes), FC-Pearson (the proposed method with the correlation $\hat {\rho }_{\text {\textit {j,k}}}$ being estimated by the Pearson’s sample correlation coefficient), and FC-Kendall (the proposed method with $\hat {\rho }_{\text {\textit {j,k}}}$ being estimated by the Kendall’s *τ*). The numbers in each cell are the mean (standard deviation) of the indicator variable for *p*-value <0.05 among the 10,000 replications

The middle panel of Table 1 compares the power of competing methods under the situation that the SNP has the same level of association with each of the phenotypic traits: **β**=(0.3,0.3,0.3,0.3,0.3)^′^. The power of MANOVA decreased rapidly as the correlation *ϱ* increased. When *ϱ*=0.75, for instance, all the other methods had the power of at least 0.4 but the power of MANOVA was only 0.2. Further, PCA and TATES had higher power than MANOVA when *ϱ*>0. Yet, none of these three methods can beat the three Fisher combination tests (FC-Permutation, FC-Pearson, and FC-Kendall) that performed equally well.

The bottom panel of Table 1 evaluates the performance of competing methods under the situation that the strength of association between the SNP and phenotypic traits varies from 0.1 to 0.5. Similar to the previous situation, the three Fisher combination tests had almost identical performance and their power decreased as the correlation *ϱ* increased. Furthermore, the Fisher combination tests had higher power than the other three methods (MANOVA, PCA, and TATES) in most conditions. MANOVA only beat the Fisher when *ϱ*=0.75; TATES had higher power than the Fisher when *ϱ*≥0.5.

Table 2 shows the simulation results when the multivariate phenotypes come from a mixture of two multivariate normal distributions. In comparison to the corresponding settings under the multivariate normal distributions in Table 1, all the competing methods tended to have lower power under these long tailed distributions. Yet, Table 2 demonstrates similar patterns to the ones observed in Table 1 in general.

**Table 2:** Simulation results when the multivariate phenotypes come from a mixture of two multivariate normal distributions

*ϱ*	MANOVA	PCA	GEE	TATES	FC-$\chi _{2m}^{2}$	FC-Permutation	FC-Pearson	FC-Kendall
	**β**=(0,0,0,0,0)^′^							
0	0.0535	0.0543	0.0135	0.0481	0.0487	0.0482	0.0461	0.0477
	(0.0023)	(0.0023)	(0.0012)	(0.0021)	(0.0022)	(0.0021)	(0.0021)	(0.0021)
0.25	0.0553	0.0514	0.0771	0.0496	0.0627	0.0465	0.0458	0.0469
	(0.0023)	(0.0022)	(0.0027)	(0.0022)	(0.0024)	(0.0021)	(0.0021)	(0.0021)
0.5	0.0537	0.0501	0.1505	0.0522	0.0895	0.0480	0.0491	0.0501
	(0.0023)	(0.0022)	(0.0036)	(0.0022)	(0.0029)	(0.0021)	(0.0022)	(0.0022)
0.75	0.0525	0.0538	0.2206	0.0481	0.1296	0.0493	0.0526	0.0513
	(0.0022)	(0.0023)	(0.0041)	(0.0021)	(0.0034)	(0.0022)	(0.0022)	(0.0022)
	**β**=(0.3,0.3,0.3,0.3,0.3)^′^							
0	0.5943	0.3299	0.8172	0.5683	0.7677	0.7633	0.7595	0.7619
	(0.0049)	(0.0047)	(0.0039)	(0.0050)	(0.0042)	(0.0043)	(0.0043)	(0.0043)
0.25	0.3038	0.5414	0.7487	0.5003	0.6779	0.6330	0.6333	0.6332
	(0.0046)	(0.0050)	(0.0043)	(0.0050)	(0.0047)	(0.0048)	(0.0048)	(0.0048)
0.5	0.2073	0.3981	0.7135	0.4402	0.6168	0.4989	0.5083	0.5082
	(0.0041)	(0.0049)	(0.0045)	(0.0050)	(0.0049)	(0.0050)	(0.0050)	(0.0050)
0.75	0.1601	0.3135	0.6847	0.3870	0.5779	0.4038	0.4111	0.4116
	(0.0037)	(0.0046)	(0.0046)	(0.0049)	(0.0049)	(0.0049)	(0.0049)	(0.0049)
	**β**=(0.1,0.2,0.3,0.4,0.5)^′^							
0	0.6972	0.4002	0.8087	0.7328	0.8451	0.8425	0.8379	0.8408
	(0.0046)	(0.0049)	(0.0039)	(0.0044)	(0.0036)	(0.0036)	(0.0037)	(0.0037)
0.25	0.4766	0.5579	0.7427	0.6698	0.7656	0.7269	0.7236	0.7259
	(0.0050)	(0.0050)	(0.0044)	(0.0047)	(0.0042)	(0.0045)	(0.0045)	(0.0045)
0.5	0.4728	0.4083	0.7073	0.6237	0.7036	0.5766	0.5855	0.5862
	(0.0050)	(0.0049)	(0.0046)	(0.0048)	(0.0046)	(0.0049)	(0.0049)	(0.0049)
0.75	0.6576	0.3172	0.6799	0.5976	0.6394	0.4532	0.4624	0.4617
	(0.0047)	(0.0047)	(0.0047)	(0.0049)	(0.0048)	(0.0050)	(0.0050)	(0.0050)

The three different effect sizes are: no effect **β**=(0,0,0,0,0)^′^; moderate effects **β**=(0.3,0.3,0.3,0.3,0.3)^′^; and varied effects **β**=(0.1,0.2,0.3,0.4,0.5)^′^. The correlation between genes is *ϱ* ranging from 0 to 0.75. The competing methods are MANOVA (Multivariate analysis of variance), PCA (Principal component analysis), GEE (Generalized estimating equations), TATES (Trait-based association test involving the extended Simes procedure), FC-$\chi _{2m}^{2}$ (the chi-squared distribution with 2*m* degrees of freedom under the independence assumption), FC-Permutation (the permutation method based on 1,000 permutes), FC-Pearson (the proposed method with the correlation $\hat {\rho }_{\text {\textit {j,k}}}$ being estimated by the Pearson’s sample correlation coefficient), and FC-Kendall (the proposed method with $\hat {\rho }_{\text {\textit {j,k}}}$ being estimated by the Kendall’s *τ*). The numbers in each cell are the mean (standard deviation) of the indicator variable for *p*-value <0.05 among the 10,000 replications

### Real data analysis

#### The Study of Addiction: Genetics and Environment(SAGE)

The National Center for Biotechnology Information (NCBI) has been managing and distributing the large database of Genotypes and Phenotypes (dbGaP) for scientific investigation of various human diseases [32]. In order to demonstrate the application of the proposed method, we conducted statistical analysis on the Study of Addiction: Genetics and Environment (SAGE) data [33], http://www.ncbi.nlm.nih.gov/projects/gap/cgi-bin/study.cgi?study_id=phs000092.v1.p1. The institutional review board of the University of Michigan has approved this secondary data analysis project (HUM00084927). The SAGE is a case-control study that aggregated together the data from three large scale studies in the substance abuse field: the Collaborative Study on the Genetics of Alcoholism (COGA), the Family Study of Cocaine Dependence (FSCD), and the Collaborative Genetic Study of Nicotine Dependence (COGEND). The total number of individuals with individual level data available is 4121. Each individual was genotyped using the Illumina Human 1M-Duo beadchip which contains over 1 million SNP markers.

We selected unrelated individuals that passed the quality control measures according to the Gene Environment Association Studies Initiative (GENEVA) quality control report. The final number of unrelated individuals is 3,741 (1,732 male, 2,079 female) and the total number of SNP markers is 917,694. Because the purpose of our analysis is to identify the genes that are associated with addiction, we used the symptomatology variables of four highly comorbid substance use disorders as the phenotype outcomes: the number of alcohol dependence symptoms endorsed (alc_sx_tot), the number of nicotine dependence symptoms endorsed (nic_sx_tot), the number of marijuana dependence symptoms endorsed (mj_sx_tot), and the number of cocaine dependence symptoms endorsed (coc_sx_tot).

#### SAGE data analysis results

**Fig. 2:**
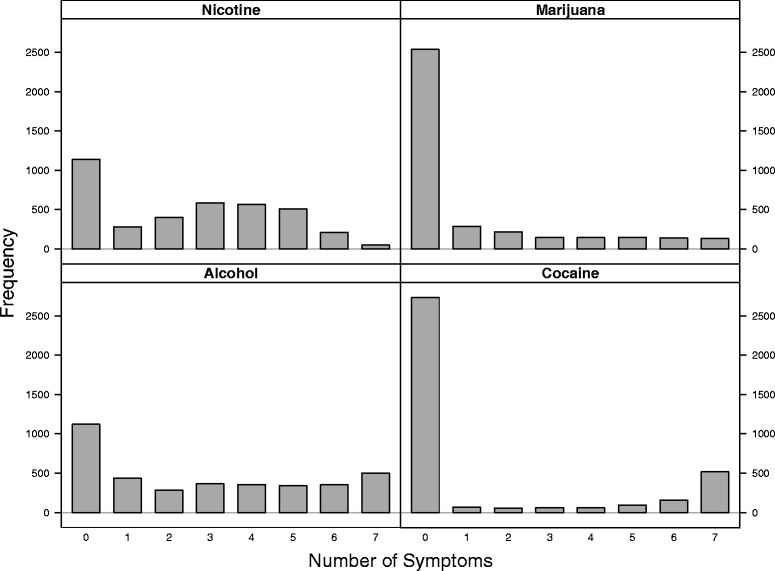
The distributions of phenotypes for alcohol, nicotine, marijuana and cocaine dependence. The *x*-axis is the number of symptoms, and the *y*-axis is the frequency

The values of phenotype variables range from 0 to 7. Figure 2 shows the frequency distributions of the 4 phenotype variables. Since they are not normally distributed, we calculated their correlations using the Kendall rank correlation. Table 3 shows moderate correlations ranged from 0.34 to 0.51.

**Table 3:** The Kendall rank pairwise correlations between alcohol, nicotine, marijuana, and cocaine outcomes

	Alcohol	Nicotine	Marijuana	Cocaine
Alcohol	1	0.4554	0.4236	0.5029
Nicotine		1	0.3373	0.3375
Marijuana			1	0.5067
Cocaine				1

**Fig. 3:**
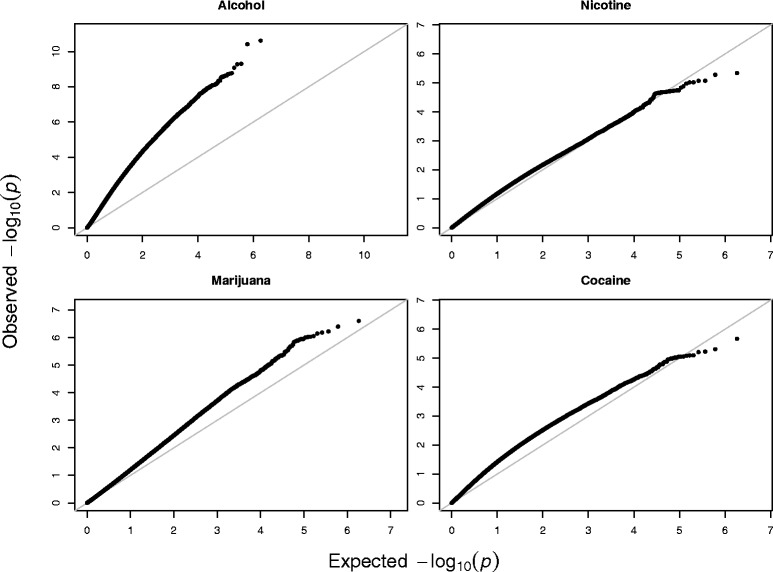
The QQ-plots of *p*-values for the marginal tests of association between the SNPs and each of the four addiction symptomatology variables. The *x*-axis is the expected −log_10_(*p*-value), and the *y*-axis is the observed −log_10_(*p*-value). The diagonal gray straight lines have the slope 1 and intercept 0

**Fig. 4:**
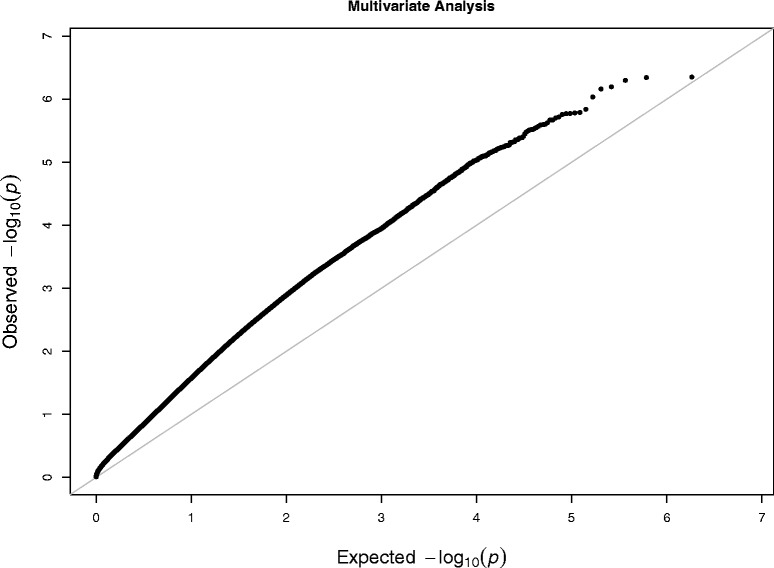
The QQ-plot of *p*-values for the Fisher combination test of association between the SNPs and the multivariate phenotype of addiction. The *x*-axis is the expected −log_10_(*p*-value), and the *y*-axis is the observed −log_10_(*p*-value). The diagonal gray straight lines have the slope 1 and intercept 0

For each trait, we conducted a genome-wide association test using the hurdle model [34] because of the discrete nature and excess zero values associated with the symptom counts. The hurdle regression model assumes the observed data are generated from two processes: one generates zero and the other generates positive values. Since our interest is in the severity of symptomatology, the *p*-values from the positive component of the hurdle model were used for further analysis. For each phenotype of addiction, the estimated *p*-values are summarized using QQ-plots in Fig. 3. The diagonal straight lines have the slope 1 and intercept 0. When the curve of the p-values deviate far away from the diagonal line, it indicates that there are many SNPs significantly associated with the corresponding phenotype trait. By examining the 4 plots in Fig. 3, we obtain the following two findings: (1) For the nicotine symptoms, the *p*-values fall on the diagonal line and this indicates that there is no SNP associated with nicotine symptoms; (2) For the symptoms of the other three substances, all the *p*-values deviate from the diagonal lines (except for 0 and 1), with the *p*-values of alcohol symptoms furthest away from the diagonal line and the *p*-values of the remaining two symptoms closer to the diagonal line. Since the four symptomatology variables are moderately correlated and they all measure the common construct of addiction, we used them as the multivariate phenotype and applied the proposed Fisher combination approach (FC-Kendall) to identify the SNPs associated with it. The QQ-plot of the *p*-values for this multivariate analysis is shown in Fig. 4 indicating that some SNPs are associated with addiction across substances.

To identify the SNPs associated with the phenotypes, we adopted the commonly used significance level for GWAS of 10^−6^ to account for multiplicity. Based on the results of the marginal tests, the numbers of SNPs identified to be associated with the individual phenotypes are 917 for alcohol dependence symptoms, 0 for nicotine symptoms, 9 for marijuana symptoms, and 0 for cocaine symptoms. Using the proposed Fisher combination method (FC-Kendall), on the other hand, we identified 6 SNPs associated with the multivariate phenotype of addiction. Among them, 5 SNPs were also identified by the marginal test for alcohol symptoms. This implies that if we ignore the correlations among the 4 phenotypic traits and conduct the marginal tests, we would identify many SNPs that may be specific to alcohol dependence. Thus, if our goal is to identify the genes associated with the construct of addiction that contributes to the 4 types of substance dependence symptomatology, the proposed method is a better approach.

## Discussion

In GWAS for complex diseases, the association between a SNP and each phenotype is usually weak. Combining multiple related phenotypic traits can increase the power of gene search and thus is a practically important area that requires methodology work. This study provides a comprehensive review of existing methods for conducting GWAS on complex diseases with multiple phenotypes including MANOVA, PCA, GEE, TATES, and the classical Fisher combination test. Built upon the Fisher combination test, we proposed a new method that relaxes the unrealistic independence assumption and is also computationally efficient. Particularly, in an exploratory study where multiple sets of phenotypes may be of interest, when the set is changed, our proposed methods only require re-calculation of the correlation between phenotypes and then the available marginal p-values for each SNP can be re-used. The competing methods which do not involve marginal *p*-values such as the PCA, MANOVA, and GEE, on the other hand, would require a complete re-analysis.

We conducted a simulation study to compare the performance of the competing methods. The GEE and the Fisher combination test with the independence assumption did not control the type I error rate and thus are not recommended. In general, the power of the methods decreased as the correlation between phenotypes increased. Furthermore, all the competing methods tended to have lower power when the multivariate phenotypes come from long tailed distributions. The proposed method (with the correlation being estimated by the Pearson’s sample correlation coefficient or the Kendall’s *τ*) performed as well as the permutation method and yet only required 10^−2^ computational time. In most settings of the simulation, these three Fisher combination tests outperformed the other methods. The real data analysis also demonstrated that the Fisher combination tests allow us to compare the marginal results with the multivariate results and specify which SNPs are specific to a particular phenotype or contribute to the common construct.

In our simulation study, we only considered continuous multivariate phenotypes. Future studies may extend the methodology work to the case of correlated discrete phenotypes. For example, in the substance abuse field, many outcomes are zero-inflated count data [35] or ordinal data [36]. A future direction that is particularly challenging is how to analyze multivariate phenotypes with different measurement scales.
